# Systemic inflammatory effects of zinc oxide particles: is a re-evaluation of exposure limits needed?

**DOI:** 10.1007/s00204-023-03567-4

**Published:** 2023-08-11

**Authors:** Christian Monsé, Rolf Merget, Jürgen Bünger, Dirk Pallapies, Thomas Brüning

**Affiliations:** Institute for Prevention and Occupational Medicine of the German Social Accident Insurance, Institute of the Ruhr-Universität Bochum (IPA), Bürkle-de-La-Camp-Platz 1, 44789 Bochum, Germany

**Keywords:** Human inhalation studies, Occupational exposure limit, Particles, Systemic inflammatory parameter, Zinc oxide

## Abstract

Exposure to airborne substances such as gases, vapours, and particles remains a relevant health risk in many workplaces. A current topic and cause for discussion is the investigation of the health effects of particles containing zinc oxide (ZnO). Among other data, those collected from our study on human exposure data of ZnO in 2018 prompted the National Research Centre for the Working Environment 2021 to formulate a new, sharply lowered proposed occupational exposure limit (OEL) for zinc in workplaces. Since the publication of the Danish report, further studies have been conducted with ZnO. In the following text, all arguments for deriving this new limit value for zinc from the report are discussed, extended with the more recent data since 2018. It should be noted that especially the application of time extrapolation factors needs further discussion and harmonization between regulatory authorities. From our point of view, the data situation can justify a higher OEL for zinc than that proposed by the Danish National Research Centre for the Working Environment.

## Introduction

Acute human inhalation studies are of great importance for risk assessment and setting of occupational exposure limits (OEL) of hazardous substances, but they are often not available or methodologically inadequate. Such studies are often performed with model substances to generate a robust basis of knowledge for a large number of hazardous substances by means of comparative observations. A current topic and cause for discussions is the investigation of health effects of particles containing zinc oxide (ZnO).

Several countries still have OELs or limit value proposals for zinc of 5 mg/m^3^ (see Table 1), although it is known that health problems such as metal fume fever can be triggered below these concentrations.

**Table 1:** OELs or limit value proposals in different countries

Country	TWA (mg/m^3^)	STEL (mg/m^3^)
Germany*	0.1	0.4
Denmark*	0.04	–
Finland	2	10 (15 min average value)
Belgium	2	10 (15 min average value)
Switzerland	0.1	0.4
USA	5	10
Hungary	5	–
New Zealand	0.1	0.5
Norway	5	–
South Korea	5	10
Latvia	0.5	–

TWA, time-weighted average is the average exposure of hazardous substances in an 8-h shift; STEL, short-term exposure limit is the acceptable average exposure over a short period of time, usually 15 min

*: Limit value proposals

In 2005, Beckett et al. compared the acute health effects of ultrafine (count median size 40 nm) with fine (count median size 260 nm) ZnO particles at an air concentration of 0.5 mg/m^3^ each. In addition, a third scenario was tested with clean air. The exposures were performed in 12 subjects with an exposure time of 2 h. No acute adverse effects could be observed, independent of the three exposure conditions (Beckett et al. 2005).

Based on the Beckett study, the German MAK Commission of the DFG proposed in 2009 a lowered limit value for zinc and its compounds of 2.0 mg/m^3^ for the inhalable fraction (E-dust) and 0.1 mg/m^3^ for the respirable fraction (A-dust) by linear extrapolation from a 2-h exposure time to an 8-h shift (DFG 2012) (0.5 mg/m^3^ ZnO A-dust for 2 h extrapolated to 8 h results in 0.125 mg/m^3^ ZnO which equals about 0.1 mg/m^3^ Zn). The derived limit value is not legally binding in Germany, since the MAK Commission only provides scientific advice to the responsible regulatory authority, but is still under discussion.

The resulting consequences of adopting the recommendation towards a legally binding OEL would be far reaching in the manufacturing industry: with "state-of-the-art" occupational hygiene, a considerable proportion of those employed in body shops would have to wear personal respiratory protection on a full shift basis. In view of the widespread processing of zinc sheet, particularly in small- and medium-sized enterprises—especially in the skilled trades—serious problems might result, since in many cases the mechanization route cannot be chosen in these areas, e.g. mechanical engineering, building and domestic services, gutters, ventilation systems, water pipes and bridge construction. The regular occurrence of higher ZnO concentrations has been confirmed by a measurement campaign carried out in 2017 in 38 hot-dip galvanizing plants in Germany. The results showed that only about 50% of the measurements near zinc boilers (personal air sampling, A-dust) were below a zinc concentration of 0.1 mg/m^3^ (Poppe et al. 2019).

An adapted human inhalation study has been proposed as a result of discussions in scientific working groups. This would make it possible to determine the lowest observed effect concentration (LOEC) to confirm the conclusions of the MAK recommendation from the Beckett et al. study or to derive a suitable occupational exposure limit for zinc. It should be noted that all considerations for conducting an inhalation study were based on a mono-exposure to ZnO, since all technical parameters can be controlled under standardized conditions. The generation of mixed exposures, as occurring especially during the welding of galvanized steel sheets, involves the risk of too many confounding factors that may not be recognized or controllable. It might be difficult to determine whether changes in medical effect parameters are due to zinc ions or to other factors.

In a previous study, we were able to demonstrate a concentration–response relationship between nano-sized ZnO particles and systemic effects (Monsé et al. 2018). Whereas no relevant effects were detectable 24 h after sham exposures and 0.5 mg/m^3^ ZnO, reversible systemic effects on acute phase proteins (C-reactive protein (CRP), serum amyloid A (SAA)) and neutrophils in blood occurred 24 h after ZnO exposures of 1.0 and 2.0 mg/m^3^ for 4 h. The effects were strongest after 2.0 mg/m^3^ ZnO, with flu-like symptoms and elevated body temperature in several subjects. In contrast to the acute phase proteins, neutrophil levels increased significantly immediately after all exposure scenarios (including sham), but not in a concentration-dependent relationship. This increase, due to physical exercise, is within the range of effect size 24 h after inhalation of 1.0 mg/m^3^ ZnO for 4 h. It is reversible and undetectable 24 h after sham exposure. It can therefore be assumed that the observable increases 24 h after ZnO inhalation were caused exclusively by ZnO. Local effects (neutrophils, interleukin-8 (IL-8), interleukin-6 (IL-6), matrix metalloproteinase (MMP-9) and tissue inhibitors of metalloproteinases (TIMP-1)) as assessed by induced sputum samples were already detectable at 0.5 mg/m^3^ ZnO, but did not show a concentration–response relationship in the range from 0.5 to 2.0 mg/m^3^ (Monsé et al. 2019). Both systemic and local effects were reversible.

The interpretation of the local effects is difficult, since no concentration–response relationship could be established, but effects were already observed at the lowest ZnO concentration, which were also present in the same strength at the highest ZnO concentration. Generally accepted reference values for the parameters are not available due to different technical study protocols. Thus, it remains unclear whether the local effects are indicative of a pathological reaction or merely represent a normal physiological response.

The lowest concentration of 0.5 mg/m^3^ ZnO did not induce fever or significant inflammatory systemic effects (Monsé et al. 2018). The study provides evidence that inhalation of nano-sized ZnO particles induces dose-dependent acute phase responses in humans at concentrations well below the current mass-based OELs in a number of countries (Table 1). Immediately after the study was released, a commentary on it was published (Vogel and Cassee 2018). This commentary suggested a causal relationship between increased levels of SAA and atherosclerosis and recommended a re-evaluation of OELs for a number of particle exposures including ZnO. Furthermore, the authors hypothesized that cardiovascular disease (CVD) might be recognized as an occupational disease.

The National Research Centre for the Working Environment (NFA 2021) in Denmark subsequently published a report in 2021 summarizing the current status on zinc and its compounds and identified our study as the only human inhalation study that examined a dose–response relationship of ZnO in detail. The working group considered chronic elevations of CRP and SAA as a relevant end point with regard to CVD, but not with regard to (acute) metal fume fever. They derived a No Observed Adverse Effect Concentration (NOAEC) from our study which showed no systemic effects after 0.5 mg/m^3^ ZnO for 4 h. After linear extrapolation to 8 h, a value of 0.25 mg/m^3^ ZnO was calculated. According to the authors, this value should be further reduced by a factor of five due to a large variation of parameters in all human studies considered and inclusion of only young and healthy subjects. According to their own statements, they followed the REACH recommendation (REACH 2018) and thus arrived at a proposed limit value of 0.05 mg/m^3^ ZnO, or 0.04 mg/m^3^ Zn, respectively.

Since the Danish zinc report was written, other papers have been published on the subject. In our second ZnO study (Monsé et al. 2021), 16 subjects were exposed to nano- and micro-sized ZnO at 2.0 mg/m^3^ and sham for 2 h each. The dose of ZnO administered (2.0 mg/m^3^ × 2 h) was derived from our first study (1.0 mg/m^3^ × 4 h) (Monsé et al. 2018) to yield mild systemic inflammatory responses and to minimize fever reactions as observed at the highest ZnO dose in our first ZnO study (2.0 mg/m^3^ × 4 h). The first ZnO study results were corroborated and it was shown that biological effects were more pronounced after exposure to micro-sized than after exposure to nano-sized ZnO particles. The stronger systemic inflammatory responses after inhalation of micro-sized ZnO particles were explained by the higher deposition efficiency in the respiratory tract by micro-sized ZnO particles. Although there was an increased effect of micro-sized ZnO particles in contrast to nano-sized ZnO in our experiments, we recommend not to consider the particle size for threshold setting because the workplace exposures remain mostly unknown. The toxicity of ZnO is driven by rapid dissolution of ZnO occurring at the low pH in lysosomes. Kim et al. found that the rapid dissolution minimizes the size dependence of the observed toxicity (Kim et al. 2016). Consequently, the Danish working group suggests using the same OEL for all ZnO particle sizes. Table 2 lists the parameters collected in our two ZnO studies. Italicized fields mark all parameters that changed after ZnO exposures. The evaluated cardiovascular data from the first ZnO study showed that exposure to ZnO did not affect blood pressure, heart rate variability and repolarization (Aweimer et al. 2020). Similarly, EBC parameters (Zn levels, pH and biomarkers) were not affected by Zn exposures (Monsé et al. 2020).

**Table 2:** Parameters collected from the IPA-ZnO studies (Aweimer et al. 2020; Monsé et al. 2018, 2019, 2020, 2021)

Body temperature	Questionnaires	Blood	Induced sputum	Cardiovascular parameters
*Time course*	*Feeling sick*	*Neutrophils*	*MMP-9*	Blood pressure
*Every 2 h*	*Feeling of fever*	*CRP*	*TIMP-1*	Heart rate variability
*Before and until 24 h*	*Muscle pain*	*SAA*	*IL-8*	Repolarization
*After exposure*	*Throat irritation*	*CC16*	*IL-6*	
	*Cough*	*IL-6*	*Neutrophils*	
	Shortness of breath	Lymphocytes	8-iso-PGF2α	
	Fatigue	Leukocytes	Substance P	
	Headache	Monocytes	Total cell number	
	Feeling warm	Thrombocytes	Total protein	
	Feeling unwell	Erythrocytes	Macrophages	
	Chills	D-dimers	Lymphocytes	
		Prothrombin F 1.2	Epithelial cells	
		Endothelial microparticles		
		Fibrinogen		

Exhaled breath condensate	FeNO	Lung function parameters	Urine	
Zinc concentration	Time course	Total lung capacity	Zinc concentration	
pH	Before, immediately	Vital capacity		
LTB4 (LTC4/ D4/E4)	After and 24 h	Residual volume		
8-iso-PGF2α	After exposure	Respiratory volume		
PGE2		Inspiratory reserve volume		
		Expiratory reserve volume		
		Functional residual capacity		
		Peak expiratory flow		
		Forced expiratory volume in 1 s		
		Tiffenau index		
		Mean expiratory flow		

Italicized fields indicate changed parameters after ZnO exposures

Inhalation studies in humans with chemically inert particles are extremely sparse in the literature; thus, it was unknown whether the effects that were observed after ZnO inhalation were due to a physical particle effect or a substance-specific characteristic. Therefore, a further acute inhalation study with the chemically inert substance barium sulphate (BaSO_4_) was conducted (Monsé et al. 2022). We selected those effect parameters that had been shown to be sensitive for the detection of airway inflammation in our previous ZnO studies (Monsé et al. 2018, 2021). Sixteen healthy subjects were exposed to micro-sized BaSO_4_ at 4.0 mg/m^3^ and sham for 2 h each. The most important result of this study was the absence of any significant differences between BaSO_4_ and sham exposures and between different time points in all parameters investigated. All systemic and local parameters whose changes occurred after ZnO inhalation were not affected in any way. These findings underline the hypothesis that the ZnO effects do not represent a physical particle effect, but a substance-specific effect. Inhaled ZnO is partially deposited in the respiratory tract. The particles are taken up in the alveoli and decomposed by macrophages for elimination from the lungs. ZnO dissolves due to a decrease of pH, releasing zinc ions in locally high concentrations. These cause the systemic and local effects that may become clinically apparent as zinc fever (Cho et al. 2011).

In summary, it is clear from these experimental ZnO inhalation studies that there is a dose–response relationship of systemic inflammatory parameters and a NOEC of 0.5 mg/m^3^ can be defined (Monsé et al. 2018). In addition, a substance-specific effect of ZnO can be assumed.

### What is the critical end point?

There is clear evidence from our studies that ZnO inhalation induces reversible systemic inflammation. This finding is corroborated by several inhalation studies which used Zn-containing welding fumes instead of pure ZnO. There is a discussion in the literature whether elevated inflammatory parameters like CRP or SAA in blood are predictive of CVD or represent only a marker of disease. In our view, a definite proof of a causal relationship between ZnO exposure and CVD would need either animal studies with ZnO or epidemiological studies in ZnO-exposed workers with the endpoint CVD. Both study types are not available. Epidemiological studies in populations with particle exposures, e.g. welders are hardly applicable because poorly soluble particles (PSP) may act via different mechanisms, including kinetics. On the other hand, the recent CANTOS study has shown that anti-inflammatory drugs can reduce CRP levels and the risk of CVD (Ridker et al. 2017). Thus, there is evidence that CRP is a predictor of CVD and is involved in the pathogenesis of CVD. It has to be remembered that not only CRP and SAA, but also neutrophils in blood increased after ZnO inhalation, which is considered a key player in the pathology of chronic pulmonary and systemic diseases (Herrero-Cervera et al. 2022). Thus, systemic inflammation due to ZnO should be regarded as “adverse”, although most authors state that there still remain important questions in the field including the mechanisms for acute phase proteins involvement, kinetics, the source, and systemic or localized expression (Shridas et al 2021; Fu et al. 2020).

### Which assessment factors should be applied?

Experimental studies may be influenced by random fluctuations and measurement inaccuracies. This was also used as an argument by the Danish working group to justify the factor of five in the calculation of the OEL for zinc (NFA 2021). We agree that there was high interindividual variation of the main systemic inflammatory parameters CRP, SAA and neutrophils in our studies. However, interindividual variation of such inflammatory parameters is not surprising. We consider random effects or measurement inaccuracies in our studies as negligible because (1) systemic inflammation was consistently shown with a battery of parameters, (2) we designed many (negative) control conditions and (3) we observed clearly negative results in our BaSO_4_ study.

A further important point for threshold setting is the transferral from young and healthy subjects to the general working population. With regard to metal fume fever, there is no epidemiologic evidence in the literature for an association between ZnO exposure and age (of the working population) or susceptibilities. For CVD, the problem is by far more complex, as many well-known risk factors for CVD exist, but their interactions with ZnO-induced systemic inflammation remain largely unknown. In our view, the transferral from young and healthy subjects to the general working population should not represent a relevant problem, especially because systemic effects were reversible.

Chronic inhalation studies in humans cannot be performed, but we consider our study design suitable to adequately represent acute inflammatory and clinical effects and to contribute to the threshold discussion. The question of “acute to chronic” exposures was investigated by Krabbe et al. 2019. They conducted an inhalation study in 15 healthy male volunteers with exposure to zinc- and copper-containing welding fumes on four consecutive days for a duration of 6 h each. The mean concentration of the welding fumes was 2.5 mg/m^3^. The welding fumes contained 60% zinc and 19.6% copper. The study showed no clinical symptoms, but a significant increase in systemic inflammatory markers in blood (CRP, SAA, and metalloprotein (MT)) throughout the simulated course of a typical welder's work week). The markers studied remained elevated at all subsequent exposures, but without cumulative effects. In that study, the airborne zinc concentration was much higher than the no observed effect concentration (NOEC) of 0.5 mg/m^3^ derived from our first ZnO study (Monsé et al. 2018). If there are no cumulative effects at high concentrations, we would not expect them at low concentrations.

In their summary the Danish working group proposed an assessment factor of five due to the large interindividual variation (NFA 2021). The German MAK Commission proposed an OEL of 0.1 mg/m^3^ for zinc in 2009, derived from the Beckett study (Beckett et al. 2005) by linear time extrapolation without an assessment factor. In our ZnO studies both clinical symptoms and systemic inflammatory effects were almost identical with 1.0 mg/m^3^ for 4 h (first study, Monsé et al. 2018) and 2.0 mg/m^3^ for 2 h (second study, Monsé et al. 2021). Our results raise the question of whether the dose or concentration is the more relevant parameter to justify a linear time extrapolation, or whether it can be dispensed with. Similar results were reported by Brand et al. (Brand et al. 2019), who showed that the main determinant of systemic inflammation induced by inhalation of ZnO and CuO containing welding fumes at higher concentration is not concentration nor duration of exposure per se but the product of both factors, total dose. The question remains whether time extrapolation is necessary at concentrations in the range of the NOEC and should be addressed with an adapted human inhalation study.

Similarly, the German MAK Commission used human inhalation studies for OEL setting of sensory irritants without an assessment factor since they are based on controlled human exposure studies assessing especially sensitive and objectively verifiable effects. As reviewed earlier these substances exert their adverse effects by (neurogenic) inflammation (Brüning et al. 2014). Thus, chronic inflammation is considered as a critical endpoint of both ZnO and sensory irritants. Although inflammation induced by sensory irritants and ZnO may be not comparable, this discussion points to the need for the harmonization of the definition of assessment factors for OELs that are based on acute human exposure studies.

## Conclusion

Our particle inhalation studies corroborate and extend the existing database of acute human inhalation studies with ZnO. The critical endpoint is chronic inflammation which is involved in the pathogenesis of CVD. An NOEC of 0.5 mg/m^3^ or higher can be derived from our studies. We believe that the application of assessment factors (for example, linear time extrapolation) requires further discussion and harmonization between regulatory authorities.

## Data Availability

The datasets generated during and/or analysed during the current studies are available from the corresponding author on reasonable request.
